# Application of synthetic photostable retinoids induces novel limb and facial phenotypes during chick embryogenesis *in vivo*

**DOI:** 10.1111/joa.12147

**Published:** 2013-12-04

**Authors:** R E Lopez-Real, J J R Budge, T B Marder, A Whiting, P N Hunt, S A Przyborski

**Affiliations:** 1 School of Biological and Biomedical Sciences, Durham UniversityDurham, UK; 2 Institut für Anorganische ChemieWürzburg, Germany; 3 Department of Chemistry, Durham UniversityDurham, UK; 4 Reinnervate Limited, NETpark IncubatorSedgefield, UK

**Keywords:** chick, craniofacial, development, limb, *Pax1*, retinoic acid, scapula, *sonic hedgehog*, synthetic retinoid

## Abstract

We have recently developed a range of synthetic retinoid analogues which include the compounds EC23 and EC19. They are stable on exposure to light and are predicted to be resistant to the normal metabolic processes involved in the inactivation of retinoids *in vivo*. Based on the position of the terminal carboxylic acid groups in the compounds we suggest that EC23 is a structural analogue of *all-trans* retinoic acid (ATRA), and EC19 is an analogue of *13-cis* retinoic acid. Their effects on the differentiation of pluripotent stem cells has been previously described *in vitro* and are consistent with this hypothesis. We present herein the first description of the effects of these molecules *in vivo*. Retinoids were applied to the anterior limb buds of chicken embryos *in ovo* via ion-exchange beads. We found that retinoid EC23 produces effects on the wing digits similar to ATRA, but does so at two orders of magnitude lower concentration. When larger quantities of EC23 are applied, a novel phenotype is obtained involving production of multiple digit 1s on the anterior limb. This corresponds to differential effects of ATRA and EC23 on *sonic hedgehog* (*shh*) expression in the developing limb bud. With EC23 application we also find digit 1 phenotypes similar to thumb duplications described in the clinical literature. EC23 and ATRA are shown to have effects on the entire proximal–distal axis of the limb, including hitherto undescribed effects on the scapula. This includes suppression of expression of the scapula marker *Pax1*. EC23 also produces effects similar to those of ATRA on the developing face, producing reductions of the upper beak at concentrations two orders of magnitude lower than ATRA. In contrast, EC19, which is structurally very similar to EC23, has novel, less severe effects on the face and rarely alters limb development. EC19 and ATRA are effective at similar concentrations. These results further demonstrate the ability of retinoids to influence embryonic development. Moreover, EC23 represents a useful new tool to investigate developmental processes and probe the mechanisms underlying congenital abnormalities in vertebrates including man.

## Introduction

Retinoids are small lipophilic molecules that are essential for a wide range of developmental events in vertebrates (Ross et al. 2000; Duester, 2008). Retinoid levels in tissues are thought to result from the balance between localised enzymatic conversion from non-biologically active retinoid precursors and inactivation by enzymes of the Cyp26 family which are also restricted during embryonic development (Swindell et al. 1999; Ross et al. 2000; Mic et al. 2004; Reijntjes et al. 2004, 2005b; Chambers et al. 2007). The restricted expression of such enzymes corresponds to embryonic regions known to be affected when retinoid levels are raised or lowered experimentally (Soprano & Soprano, 1995; Ross et al. 2000; Yashiro et al. 2004). This suggests that retinoids are required for selected embryonic processes, and that correct retinoid concentration and localisation are necessary.

A major part of retinoid effects are mediated by the binding of *all-trans* retinoic acid (ATRA) to retinoic acid receptors (RARs), which results in modulation in the transcriptional activity of a range of target genes (Mark et al. 2006). However, there is evidence that retinoids other than ATRA can also exert biological effects. It is known that two isomers of ATRA, *9-cis* and *13-cis* retinoic acid, can be found *in vivo*. When a teratogenic dose of ATRA was administered to e10.5 mouse embryos, a considerable increase in the levels of *13-cis* retinoic acid was detected in the spinal cord, one of the embryonic regions subject to retinoid teratogenesis (Horton & Maden, 1995). The isomeric *9-cis* retinoic acid can be produced by photo-conversion of ATRA and is approximately 25 times more potent than ATRA at inducing digit duplications in chick limbs (Thaller et al. 1993). Oxidation of retinoids by the CYP26 enzymes is generally thought to result in their inactivation (White et al. 1996, 2000; Fujii et al. 1997). However, the abnormalities in vitamin A-deficient quail embryos can be rescued to a large extent by administration of metabolites 4-oxo retinoic acid, 5,6-epoxy retinoic acid or 4-hydroxy retinoic acid (Reijntjes et al. 2005a). Thus, it is possible that some effects of retinoids may be mediated by ATRA metabolites.

There is extensive literature concerning the ability of retinoids to influence a number of systems *in vivo*. One of the best studied retinoid sensitive systems is the developing forelimb bud, where early work demonstrated that retinoids are able to influence patterning along the anterior–posterior axis (Tickle et al. 1982). In this system retinoids can be applied topically to the anterior limb bud using ion-exchange beads soaked in retinoid-containing solutions. It was found that additional posterior digits on the anterior side of the limb were produced, and the number and identity of the digits developing were proportional to the concentration of retinoid supplied to the limb (Tickle et al. 1985). Thus, naturally occurring retinoids are able to produce a dose-dependent duplication and posteriorisation of digit pattern.

The three digits in the avian wing have been described as corresponding to digits 2, 3 and 4 (Burke & Feduccia, 1997). However, fossil evidence would suggest that digits of the bird wing correspond to digits 1, 2 and 3 in other tetrapods (Burke & Feduccia, 1997; Towers et al. 2011). The recent fate mapping of the contribution of the polarising region to the digit skeleton (Towers et al. 2011) and the transcriptional profiling of digit precursors in chick forelimbs and hindlimbs (Wang et al. 2011) have added further evidence supporting this concept. The nomenclature used throughout this work when referring to wing digits of different identities will reflect these findings.

Endogenous retinoids are required for the establishment of the proximal part of the forelimb field (Stratford et al. 1996; Niederreither et al. 2002; Mic et al. 2004; Yashiro et al. 2004; Zhao et al. 2009; Cunningham et al. 2013). The ability of topical retinoid application to induce duplication and posteriorisation of digits is thought to result from their ability to induce *Hand2* and *shh* expression (Riddle et al. 1993; Fernandez-Teran et al. 2000) and does not reflect a direct endogenous role for retinoids in antero-posterior patterning of the digits. Nevertheless, the graded nature of the response of limb buds to retinoids means that the limb continues to represent a sensitive system for the comparison of the biological effects of retinoids *in vivo* (Tickle et al. 1985).

There is considerable interest in using retinoids to investigate developmental mechanisms and to control cell differentiation *in vitro*, e.g. neurogenic differentiation of embryonal carcinoma stem cells (Przyborski et al. 2000; Horrocks et al. 2003; Stewart et al. 2003). However, a limitation in the use of naturally occurring retinoids is that they can give rise to a range of different products upon exposure to light (Christie et al. 2008). This will deplete the quantity of defined retinoid present and generate a mixture of compounds that are also biologically active but with potentially different properties to ATRA (Thaller et al. 1993; Murayama et al. 1997). From the perspective of experimental reproducibility, a compound with similar properties to ATRA but without its instability to light, or to other environmental conditions, would offer considerable advantages.

**Figure 1:**
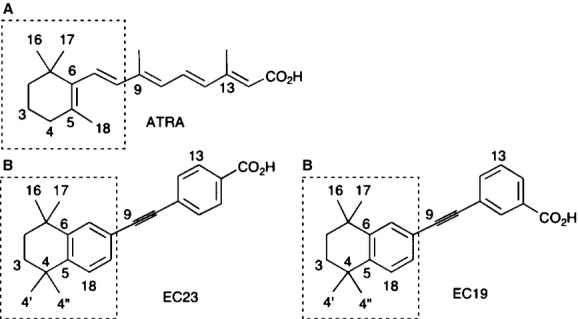
Structures of synthetic retinoids used in this study compared with the naturally occurring *all-trans* retinoic acid (ATRA). The two synthetic retinoids, EC19 and EC23, differ only in the positioning of the terminal carboxylic acid group. Note that non-IUPAC numbering is used to highlight selected carbon atoms which map similarly onto each of the structures. The numbered carbons are those referred to at some point in the text. (A) Trimethylcyclohexenyl ring and part of the polyene chain of ATRA. (B) Tetramethyl-tetrahydronaphthalene unit which forms the equivalent part of the two synthetic retinoids. The terminal carboxylic acid groups are shown as CO_2_H.

We have recently developed a range of retinoid analogues which have been demonstrated to be stable on exposure to light (Christie et al. 2008). In addition, they would be predicted to be largely resistant to oxidation by cyp26 enzymes on the basis of their structure (see Discussion herein). Two of these compounds have been demonstrated to promote differentiation of TERA2.cl.SP12 embryonal carcinoma stem cell lines *in vitro* (Christie et al. 2008) and are illustrated in Fig. 1. The two synthetic compounds differ only in the position of the terminal carboxylic acid group with respect to the tetramethyl-tetrahydronaphthalene unit that replaces the trimethylcyclohexenyl ring and part of the polyene chain found in naturally occurring retinoids (see boxes in Fig. 1 and the structure numbering, highlighting which carbon atoms map onto which, on each structure). The position of the terminal carboxlic acid suggests that EC23 would be a very close analogue of ATRA, as reinforced by the fact that the two structures are essentially superimposable. In contrast, EC19 would be a closer analogue of *13-cis* retinoic acid. Consequently, EC23 was able to promote neurogenic differentiation in culture efficiently, like ATRA, although EC23 was significantly more potent. In contrast, EC19 produced epithelioid differentiation characterised by enhanced cytokeratin expression (Christie et al. 2008).

This shows that one synthetic retinoid can give rise to similar effects as those resulting from ATRA exposure. To characterise further these compounds we have explored their properties *in vivo*. As discussed above, the effects of retinoids on limb digit identity are dose-dependent. In light of the *in vitro* evidence that they are more potent, we have chosen to use the chick embryonic limb bud system to evaluate the effects of these novel retinoids directly on vertebrate embryogenesis.

## Methods

Fertile white leghorn eggs were obtained from PD Hook Hatcheries (Thirsk, North Yorkshire). They were kept at 10 °C and then incubated at 38.4 °C in 50% humidity. *All-trans* retinoic acid (ATRA; Sigma) was dissolved in dimethyl sulphoxide (DMSO; Sigma). EC23 and EC19 were obtained from Reinnervate Ltd and dissolved in DMSO. Aliquots of ATRA, EC23 and EC19 were stored at −20 °C.

### *In ovo* microsurgery

Eggs were incubated for 4 days and staged according to Hamburger and Hamilton (Hamburger & Hamilton 1951) and Fisher et al. (2008). Eggs staged at HH20–21 were then treated with retinoid or DMSO. AG1-×2 beads (diameter 50–150 or 150–300 μm; BioRad) were used as formate derivatives, prepared from chloride or hydroxide forms according to manufacturer's instructions. Beads were soaked in retinoid or DMSO for 30 min, rinsed in Tyrode's saline (Tickle et al. 1985) and then inserted into a slit in the anterior limb bud using fine forceps and tungsten needles. Eggs were re-incubated for 7 days for phenotypic screens of the retinoids, 24 h for RNA analysis, and 30 or 48 h for *in situ* hybridisation.

### Whole-mount Alcian Blue staining and photography

Embryos recovered after 7 days of incubation with retinoid were then stained with Alcian Blue and Alizarin Red to determine their skeletal phenotype. Briefly, embryos were fixed and stained in ethanolic acetic Alcian Blue for at least 3 days, followed by three washes in 100% ethanol over 3 days. Embryos were imaged for limb and beak malformations using a Spot Idea camera mounted on a Zeiss SV11 dissecting microscope. Embryos were imaged dry or surrounded by 100% ethanol on 3% agar plates. To allow for comparisons of beak length, the left eye was removed to allow the heads to lie flat. Embryos were then stained with Alizarin Red in 1% aqueous potassium hydroxide for 45 min to stain for bone. Embryos were cleared in 1% potassium hydroxide (KOH). Embryos were then further cleared in 1% KOH, 25% glycerol and stored in 1% KOH, 50% glycerol or 80% glycerol. Embryos were then imaged using transmitted light.

### Assignment of digit identity

To establish a set of criteria which could be used to identify digits we measured their length in images of 19 control limbs using the line segment function of NIH IMAGEJ. From these measurements we calculated the following length ratios with respect to digit 2:

1. digit 1 – 0.42, 0.05 S.D.
2. digit 2 – 1.00
3. digit 3 – 0.58, 0.05 S.D.

The crucial distinction to be made for our purposes was between digit 1 and digit 2. Based on the above measurements we interpreted a duplicated digit as digit 1 if the length ratio was < 0.5.

### Reverse transcription

Chick RNA was reverse-transcribed using the High Capacity DNA Synthesis kit (Applied Biosystems, Paisley, UK) as per the instructions provided. Briefly, 2 μg RNA in 10 μL was added to 1 μL 10× buffer, 0.4 μL dNTP, 1 μL random primers, 1 μL reverse transcriptase and nuclease-free water (Promega, Southampton, UK). This was then placed into a thermocycler (Biometra) with the following programme: 25 °C 10 min, 2 h at 37 °C and 85 °C 10 s.

### Polymerase chain reaction

To generate polymerase chain reaction (PCR) products which could in turn be transcribed into labelled RNA probes, a T7 RNA polymerase binding site was added to the 5′ of each reverse primer. Primers were designed using sequences from the National Centre for Biotechnology Information (NCBI) and are shown below. Before designing primers which would generate specific probes, the sequence of interest was blast-searched to determine regions of homology and specificity. The probes which would be generated for each gene were then blast-searched to ensure specificity. For primer sequences see Table 1 below. PCR was carried out using GoTaq Flexi DNA Polymerase kit (Promega) according to the manufacturer's instructions.

**Table 1:** Sequences used to generate primers for PCR and subsequent whole-mount *in situ* hybridisation.

U22046.1 shh (Riddle et al. 1993)	TGGAGCAGACGGGTGGGTA	TTCCTCGGCGGCTTTGTC
NM_204821.1 Pax1 (Barnes et al. 1996)	ATGAAGAGAACACGGGAGCTGACA	TCCTGATTTCGCTGCCACTGAGTT
T7 polymerase	AAGGATCCGTCGACATCGATAATACGACTCACTATAAGGGA	

### Probe synthesis

Digoxigenin-labelled (DIG-labelled) RNA probes were generated from total RNA from HH20 embryos. RNA was extracted and reverse-transcribed as described earlier. The complementary DNA (cDNA) was used as a template for PCR to amplify the region of DNA specific to each probe. The PCR product was then used as a template for *in vitro* transcription of DIG-labelled RNA probes for whole-mount *in situ* hybridisation. The PCR fragments were transcribed into DIG-labelled RNA. A 1 μL template was added to 2 μL buffer, 0.2 m dithiothreitol, 2 μL nucleotides containing DIG-uracil triphosphate (Roche Applied Science; Burgess Hill, UK), 0.5 μL recombinant ribonuclease inhibitor (RNasin) and 1 μL T7 RNA polymerase (Promega) in 20 μL reaction volume. This was incubated at 37–45 °C for 2 h and analysed on 2% agarose tris-borate EDTA (TBE; Sigma) gel. The size of the product was checked against a low range DNA ladder (Fermentas). The probe was then ethanol-precipitated and washed twice with 80% ethanol before allowing it to dry in the air. The probe was then re-suspended in 1×TE for quantification on the NanoDrop spectrophotometer (ND1000).

### Prehybridisation, post-hybridisation and visualisation

Whole-mount *in situ* hybridisation was carried out according to Acloque et al. (2008) with some modifications. Embryos were incubated to the desired stage. They were dissected into ice-cold phosphate-buffered saline (PBS) and fixed overnight in 4% paraformaldehyde (Sigma) prepared in diethylpyrocarbonate (DEPC)-treated PBS at 4 °C. After washing three times in DEPC-PBS containing 0.1% Tween20 (Sigma; PBT), membranes were dissected off before dehydrating to 100% methanol (AnalaR VWR; Lutterworth, UK). Batches were stored in 100% methanol at −20 °C. They were rehydrated to PBT and then bleached in 6% hydrogen peroxide (Sigma). This was rinsed off with PBT twice before digestion with proteinase K (Sigma) : PBT to allow the probe access. Proteinase K was applied at 10 or 20 μg mL^−1^ for 3-or 4-day-old embryos, respectively, for 15 min. This enzyme was blocked using 2 mg mL^−1^ glycine (AnalaR VWR) and rinsed with PBT. Embryos were re-fixed in 0.2% glutaradehyde : 4% paraformaldehyde : DEPC-PBS for 20 min followed by PBT. Embryos were then treated with 0.1 m triethanolamine hydrochloride (pH 8) and 0.25% (v/v) acetic anhydride (Sigma) to reduce background. Hybridisation was carried out using highly stringent conditions. Embryos were equilibrated to the hybridisation solution and the temperature via three washes: first a 1 : 1 prehybridisation : PBT wash, followed by a wash in 100% prehybridisation solution at room temperature and one wash in 100% prehybridisation solution at 70 °C for 1 h. Approximately 1 μg DIG-labelled RNA probe was then dissolved into 1 mL prehybridisation buffer in which embryos were agitated at 70 °C for 16 h.

Embryos were then washed with solution 1 at 70 °C (50% formamide, 5×SSC, 1% sodium dodecyl sulphate, SDS) and then with solution 3 (50% formamide, 2×SSC, 1% SDS) at 70 °C. This was followed by TBST washes [1×TBS, 1% Tween20, 2 mm levamisole (Sigma)] and then blocking with 10% sheep serum (Sigma) : TBST. Anti-DIG antibody 1 μL (Roche Applied Science) was purified using embryo powder for 1 h in 1% sheep serum TBST at 4 °C. The powder was then centrifuged to remove purified antibody in the supernatant and was applied to the embryos at 1 : 3000 dilution. This was rocked overnight at 4 °C.

Antibody was removed and followed with eight washes of TBST and left overnight at 4 °C. The embryos were then placed in NTMT (100 mm sodium chloride, 100 mm TrisHCl pH 9.5, 50 mm magnesium chloride, 0.1% Tween20, 2 mm levamisole). The DIG-antibody was conjugated to alkaline phosphatase, which will catalyse a reaction between nitro-blue tetrazolium chloride (NBT) and 5-bromo-4-chloro-3-indolylphosphate toluidine salt (BCIP) to produce a purple precipitate. NBT 4.5 μL mL^−1^ and BCIP 3.5 μL mL^−1^ (Promega) were applied with NTMT and agitated in the dark for 20 min and left to develop. Once the colour reaction had developed to the desired extent, embryos were washed three times with PBS and then fixed in 4% paraformaldehyde. Embryos were then imaged using the Idea (Spot) camera on the Zeiss SV11 dissecting microscope as described previously. Embryos were imaged on 3% agar plates so that they could be flat and pinned in place.

### Embryo dissection, RNA isolation and qPCR

Embryos were exposed to retinoid or DMSO as described above by *in ovo* microsurgery. Twenty-four hours after operating, embryos were dissected into ice-cold DEPC-PBS. Embryos whose limbs still had a bead attached and had developed as expected were included in the pool for RNA isolation. Torsos of embryos were dissected and pinned to 3% agar : Tyrode plates to facilitate accurate dissection. The bead was removed and the anterior third of the limb bud was dissected using tungsten needles. The anterior limb portion was transferred into an RNase free tube (Starlabs) in DEPC-PBS. After dissection, DEPC-PBS was removed and RLT (lysis buffer) was then placed on the limb portions. The limb portions were lysed and homogenised. Limb portion lysates were stored at −80 °C until 16 limb portions were collected per repeat. They were then thawed on ice and RNA was isolated using the Qiagen RNeasy Kit (Dorking, UK) as per the manufacturer's instructions. RNA was quantified using the nanodrop ND1000.

Reverse transcription was carried out using cDNA generated using the high capacity cDNA synthesis kit described earlier. qPCR was carried out using the Applied Biosystems Real Time PCR 7500 System as per the manufacturer's kits and instructions. The genes analysed and the assay IDs (Taqman Gene Expression Assays; Applied Biosystems) were the following:

*cyp26A1* Gg03345448_g1
*Raldh2* Gg03348020_m1
*Meis2* Gg03338704_m1

Briefly, 20 ng of DNA was used per qPCR reaction, which was carried out as per the manufacturer's instructions.

In all cases no expression was detected in the blank reaction. Change in gene expression was calculated as fold change by the ΔΔ*C*_*t*_ method using Gapdh expression as the endogenous control. The relative quantification (RQ) was then calculated using the formula: RQ = 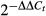, and standard deviations were then calculated for these values. The significance of the fold change in response to retinoid with respect to DMSO was calculated using an unpaired Student's *t*-test.

## Results

### The response of embryos to synthetic retinoids

Our initial experiments compared the toxicity of synthetic retinoids EC23 and EC19, and naturally occurring ATRA. AG1-X2 resin beads of 200–400 mesh size, diameter 50–150 μm (classed as small beads), were soaked in retinoid-containing solutions of different concentration and implanted into anterior limb buds of chick embryos at Hamburger and Hamilton stage 20 (HH20) (Hamburger & Hamilton, 1951). Embryos were incubated until HH35, and the number surviving to this stage scored. The results are shown in Table 2. It can be seen that EC23 is the most toxic, with no embryos surviving applications of beads soaked in a concentration of retinoid higher than 0.01 mg mL^−1^. In contrast, the toxicity of both EC19 and ATRA was similar to that described in the previous literature for ATRA (Tamarin et al. 1984; Tickle et al. 1985), with embryos surviving applications of beads soaked in concentrations up to 10 mg mL^−1^ ATRA. EC23 is thus toxic at three orders of magnitude lower concentration than ATRA. The synthetic retinoid EC19 exhibited the unusual characteristic of showing increased toxicity compared with the naturally occurring ATRA at 0.01 mg mL^−1^, but less toxicity than ATRA at 1 and 3 mg mL^−1^.

**Table 2:** Embryo survival rate through to stage HH35 (Hamburger & Hamilton, 1951) after receiving implants to the anterior limb bud of 50–150-μm diameter resin beads soaked in different concentrations of retinoid. The data show the number of operations carried out at the retinoid concentrations used and the percentage survival rate.

Concentration (mg mL^−1^ in DMSO)	Number of operations conducted	Percentage (number) of embryos surviving up to stage 35
EC23		
0.0001	32	75 (24)
0.001	70	36 (25)
0.01	57	53 (30)
0.1	31	0 (0)
EC19		
0.01	26	23 (6)
0.1	19	47 (9)
1	25	80 (20)
3	31	68 (21)
ATRA		
0.01	135	31 (42)
0.1	47	30 (14)
1	24	38 (9)
3	27	26 (7)
10	22	50 (11)

**Figure 2:**
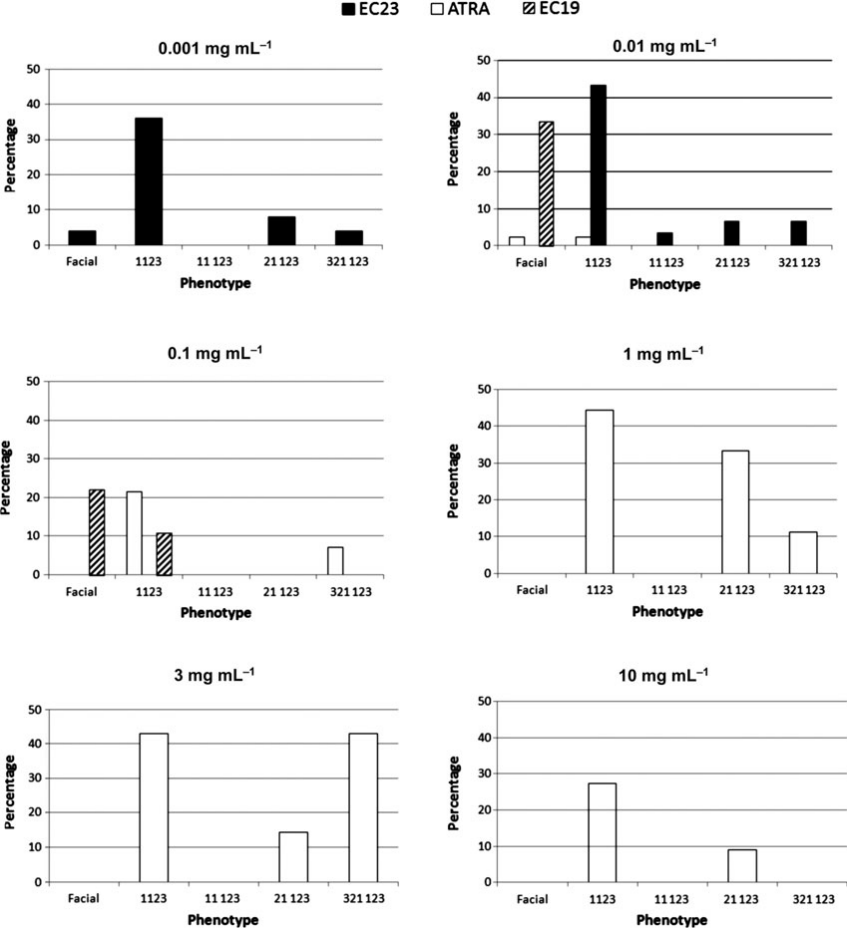
Incidence of limb and facial phenotypes after treatment with retinoid-soaked resin beads of 50–150 μm diameter. The concentration of retinoids used is shown above each panel. Data are shown for all treatments that were attempted, and represent the percentage of embryos in each treatment group surviving to stage 35 with the indicated phenotypes. For the 0.1 mg mL^−1^ treatment class there was no survival to stage 35 of embryos treated with this dose of EC23. The phenotype numbers refer to the pattern of wing digits that arose in the treated limbs. The normal wing digit pattern is 123. 1123 represents an additional digit 1 and 11123 represents two additional digit 1.

**Figure 3:**
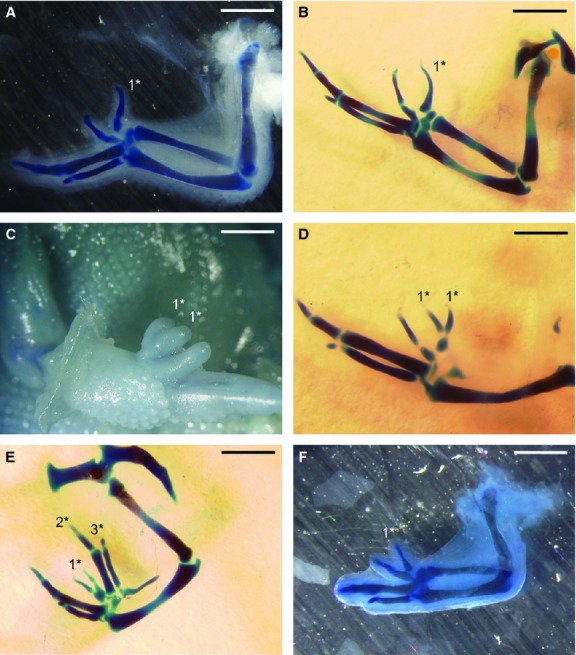
The range of limb phenotypes seen after treatment with novel synthetic retinoids via beads of 50–150 μm diameter. Additional digits are indicated with a number corresponding to the digit's identity followed by an asterisk. (A,B) Digit 1 duplications (1123 phenotype) after exposure to 0.001 and 0.01 mg mL^−1^ EC23, respectively. Length ratios of the anterior 2 digits were 0.33 (1*)and 0.39 (1). (C,D) A multiple digit 1 duplication (11123 phenotype) after treatment with 0.01 mg mL^−1^ EC23. (C,D) Images of the same specimen before and after clearing, respectively. Length ratios of the anterior 3 digits were 0.31 (1*), 0.19 (1*) and 0.40. (E) Duplication of digits 3, 2 and 1 (321123 phenotype) after treatment with 0.01 mg mL^−1^ EC23. A bead is visible, embedded in the scapula in this image. Length ratios for the additional digits were 0.56 (3*), 0.94 (2*)and 0.38 (1*). (F) Digit 1 duplication (1123 phenotype) obtained with 3 mg mL^−1^ EC19. Scale bars: 2 mm.

The naturally occurring retinoid ATRA is known to produce digit duplications (Tickle et al. 1982). In addition to this, topical application of ATRA to the limb has been shown to produce selective reduction in the formation of the upper beak (Tamarin et al. 1984). This may be either because of proximity of the limb bud to the facial primordial *in ovo* or because the retinoid levels in the entire embryo are systemically raised after topical application to the limb (Tickle et al. 1985). The limb bud and its vicinity are already extensively vascularised at the time when beads are applied (Vargesson, 2003; Therapontos et al. 2009). This vasculature could potentially act as a route by which other parts of the embryo, including the face, could be exposed to retinoid. The two synthetic retinoids differed in their ability to induce either of these biological effects. The relative frequencies of different limb and facial phenotypes over a range of different retinoid concentrations are shown in Fig. 2. Overall EC23 was similar to ATRA in the spectrum of digit duplications it produced, but was able to do so at a 10–100-fold lower concentration. EC23 produced a full spectrum of digit duplications at 0.001 and 0.01 mg mL^−1^, whereas ATRA could only produce the mildest form of digit duplication, a duplicated digit 1, at 0.01 mg mL^−1^. Figure 3(A,B) shows duplications of digit 1 obtained with 0.001 and 0.01 mg mL^−1^ EC23, respectively. Figure 3(E) shows duplications of digits 3, 2 and 1 with 0.01 mg mL^−1^ EC23. Higher doses of ATRA could produce the full spectrum of digit duplications previously described (Tickle et al. 1982). In addition, EC23 was able to produce a novel type of digit duplication not previously described after ATRA treatment. This was obtained after treatment with 0.01 mg mL^−1^ EC23, and involves multiple duplications of digit 1, in the absence of the formation of any other types of duplicated digit. Figure 3(C,D) shows this digit duplication phenotype, which is labelled as 11123 in Fig. 2. EC19 produced duplications of a single digit 1 at low frequency. It is notable that much higher doses of EC19 were required to obtain this phenotype compared with EC23, even though both retinoids have been shown to be stable. This is consistent with evidence of their potency *in vitro* (Christie et al. 2008). Thus, we have found that these synthetic retinoids can have similar effects to naturally occurring retinoids on the developing limb. However, there are some differences in their toxicity and effective dose compared with naturally occurring retinoids. In addition, we have found that the most potent novel retinoid, EC23, produces a novel phenotype of multiple digit 1 duplications that has not been previously described after ATRA treatment.

### The response of embryos to larger quantities of synthetic retinoids

Our toxicity data indicate that increasing the concentration of EC23 to 0.1 mg mL^−1^ prevented any embryos surviving to HH35. We wished to investigate the phenomenon of the multiple digit 1 duplications further. We therefore investigated the consequences of applying a larger quantity of retinoid to limb buds at the same maximum concentration found to be compatible with embryo survival. To do this we used ion exchange beads of 50–100 mesh size, with diameters of 150–300 μm (classed as large beads). As a consequence of their size, these beads have four times the surface area of the smaller beads used in our initial experiments.

**Figure 4:**
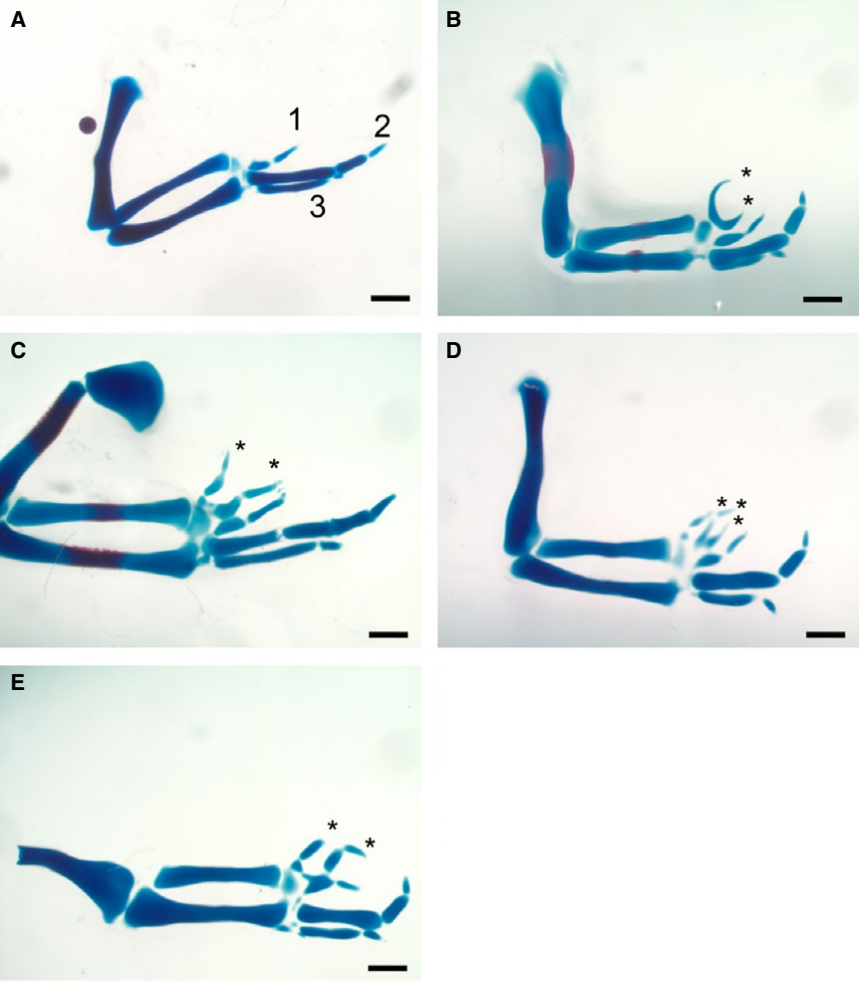
The effect of application of 0.01 mg mL^−1^ EC23 on chick wing development via beads of 150–300 μm diameter. Dorsal views of whole mount skeletal preparations of isolated retinoid treated wings are shown. (A) Effect of DMSO with normal digit development shown by 123. (B-E) Examples of multiple digit 1 duplications generated with EC23. Additional digit 1s are highlighted by asterisks. Length ratios of digits anterior of digit 2 are 0.44, 0.23 and 0.48 (B), 0.26, 0.38 and 0.40 (C), 0.27, 0.41, 0.25 and 0.45 (D) and 0.37, 0.59 and 0.49 (E). Panel E may represent a unique example of a 12123 duplication induced by EC23. Scale bars: 1 mm.

The survival rate of embryos to HH35 when treated with the larger beads is shown in Table 3. The outcomes are similar to those observed with smaller beads soaked in similar concentrations of retinoid shown in Table 2. Survival rates with EC23 at 0.01 mg mL^−1^ are similar to those seen with smaller beads, and the increased survival of embryos treated with 0.1 mg mL^−1^ EC19 compared with 0.01 mg mL^−1^ EC19 is confirmed. This is a relatively low survival rate despite administration of retinoid to the limb, a structure that is not required for embryonic survival. This may reflect that the limb is sufficiently well vascularised at this stage for retinoids to be transferred to other, more vital structures, such as the heart. The phenotypes resulting from treatment with this quantity of retinoid are shown in Table 4. With an increased quantity of EC23, it is notable that embryos either have no alterations to the digit pattern or exhibit an additional or multiple digit 1s. Duplications of digits 2 and 3 are rarely seen with this quantity of EC23. In contrast, increasing the quantity of 1 mg mL^−1^ ATRA delivered still resulted in duplication of posterior digits in addition to digit 1, as shown in Table 4. This indicates that the change in bead size alone is unlikely to be causing the formation of multiple digit 1s. ATRA application is only able to result in the duplication of one additional digit 1, whereas multiple digit 1 duplications are the outcome specifically caused by the novel stable retinoid EC23, as shown in Fig. 4. What is also notable is that we found no cases after EC23 treatment in which more than three additional digit 1s were formed. In Fig. 4(E) the central additional digit has a length ratio of 0.59, putting it outside the range of digit 1. This may represent a unique example of an additional digit intermediate in identity between digit 1 and digit 2. Delivery of an increased quantity of EC19 did not result in any observed digit duplications (*n* = 16) even at 0.1 mg mL^−1^ (*n* = 10), which produced digit duplications with small beads (Figure 2, data not shown).

**Table 3:** Toxicity of the synthetic retinoids EC23 and EC19 compared with ATRA when applied on beads of 150–300 μm diameter. This shows the number of operations carried out at the retinoid concentrations used. This is used to calculate the frequency/% survival 7 days after operation. % survival is presented as a percentage (number of embryos).

Retinoid concentration (mg mL^−1^ in DMSO)	Number of operations			% survival (number) surviving to 7 days		
	ATRA	EC23	EC19	ATRA	EC23	EC19
0.01	0	58	20	–	50 (29)	30 (6)
0.1	0	0	24	–	–	42 (10)
1	14	0	–	43 (6)	–	–

**Table 4:** Frequency and nature of limb phenotypes generated with ATRA and EC23 applied on beads with diameter of 150–300 μm. Normal limb development is referred to as 123. Extra digits are denoted by numbers before this and refer to the digit identity assigned by length of ectopic digits.

Retinoid	Concentration (mg mL^−1^)	Frequency (number) of limb phenotypes seen in embryos surviving to 7 days							
		123	1123	11 123	111 123	2123	21 123	1223	32 123
EC23	0.01	35 (10)	24 (7)	17 (5)	3 (1)	0	0	0	0
ATRA	1	0 (0)	33 (2)	0	0	17(1)	17 (1)	17 (1)	17 (1)

### Limb buds are responding to added retinoid at the transcriptional level

**Figure 5:**
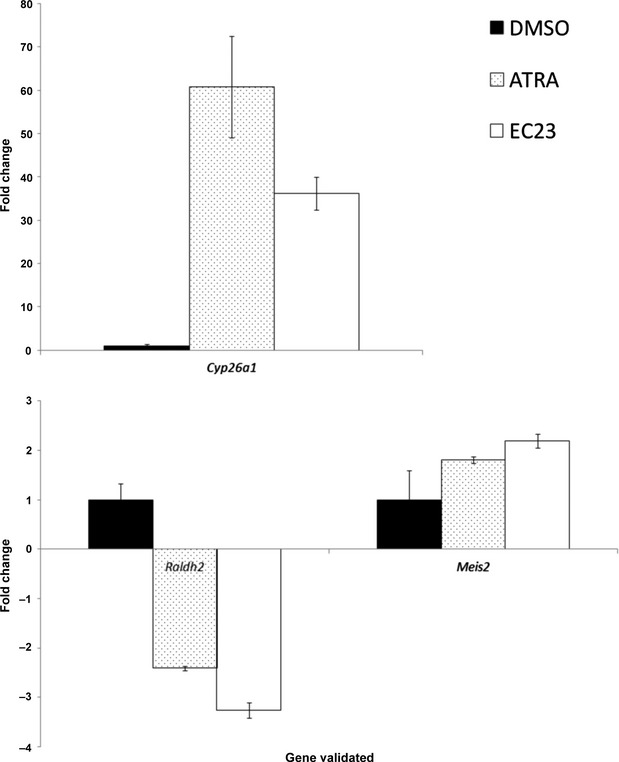
Expression of retinoid target genes after ATRA or EC23 treatment by qPCR. Fold change is with respect to DMSO-treated wing buds. Level of expression in DMSO scaled to 1 for each gene. *n* = 3, error bars ± SD.

Retinoids are known to exert their effects by a range of cellular mechanisms. Most effects in developing systems are assumed to be mediated by modulation of transcription, and a large number of genes have been described as regulatory targets of retinoids (Balmer & Blomhoff, 2002). Two genes known to be retinoid-regulated are *Cyp26a1*(Reijntjes et al. 2005a) and *Raldh2*, an enzyme that converts retinaldehyde to the biologically active retinoic acid (Niederreither et al. 1999). *Cyp26a1* is retinoid-inducible, whereas *Raldh2* expression is suppressed by retinoids. A third gene, *Meis2*, is known to be a proximal limb marker and is upregulated by ATRA (Mercader et al. 2000). We used qPCR to investigate whether EC23 was likely to be exerting its effects via transcription. We compared the expression of these three known retinoid target genes in limb buds 24 h after treatment with DMSO and retinoids. Figure 5 shows that expression of *Cyp26a1* is induced 60-fold by ATRA and 35-fold by EC23. In contrast expression of *Raldh2* is reduced 2.5-fold by ATRA and 3.25-fold by EC23. This is consistent with the previously described response of these genes to retinoids, and suggests that the effects of EC23 are mediated transcriptionally. It also suggests that the system is exhibiting feedback inhibition in response to excess retinoid. Increased *Cyp26* levels would increase the rate of breakdown of retinoids susceptible to metabolism, whereas reduced levels of *Raldh2* would reduce levels of ATRA synthesis. Levels of the marker of proximal wings, *Meis2*, are induced 2-fold and 2.5-fold by ATRA and EC23, respectively. This is consistent with earlier work showing this gene is retinoid-inducible, and that cells are being proximalised as a result of retinoid treatment (Mercader et al. 2000).

### The effect of retinoids on Shh expression

**Figure 6:**
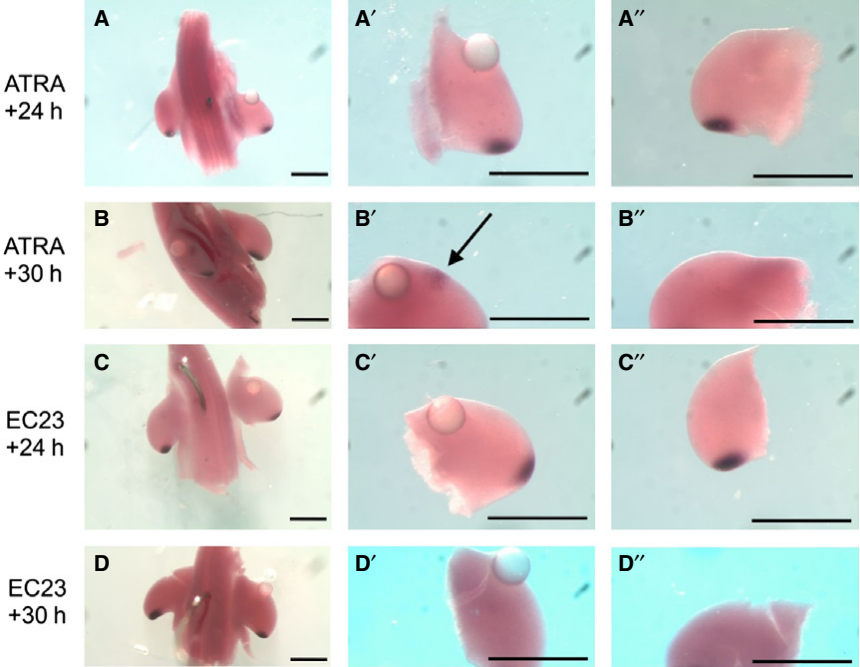
Expression of *shh* after treatment of wing buds at HH20 with 1 mg mL^−1^ ATRA or 0.01 mg mL^−1^ EC23. (A,B) *shh* expression 24 and 30 h, respectively, after treatment with ATRA. (C,D) illustrate *shh* expression 24 and 30 h respectively after treatment with 0.01 mg mL^−1^ EC23. (A-D) Intact torsos of the treated embryos. (A′-D′) Retinoid-treated limb buds after isolation; (A″-D″) corresponding isolated contralateral (control) wing buds. The arrow in (B′) highlights ectopic *shh* expression detected 30 h after treatment with 1 mg mL^−1^ ATRA. Scale bars: 1 mm.

Previous work has shown that retinoid-induced digit duplications are associated with the induction of ectopic, anterior domains of *shh* expression after 24–30 h of retinoid treatment (Riddle et al. 1993). Furthermore, ectopic *shh* expression alone is able to induce digit duplications (Riddle et al. 1993; Chang et al. 1994). The expression of *shh* after retinoid treatment was therefore investigated by whole-mount *in situ* hybridisation. Figure 6(A,C) shows that after 24 h treatment with either ATRA or EC23 no ectopic *shh* domain is observed on the anterior wing bud after retinoid treatment. However, as seen in Fig. 6(B), ATRA was able to induce *shh* expression in the anterior wing bud at a similar frequency to its ability to produce severe digit duplications (*n* = 2/6) after 30 h of retinoid treatment. EC23 was never observed to induce *shh* expression in the anterior wing (*n* = 11) as shown in Fig. 6D. This difference in *shh* induction suggests that digit duplication and/or limb development is inhibited to a greater extent in EC23-treated wings.

### Synthetic retinoids are able to affect proximal limb elements

**Figure 7:**
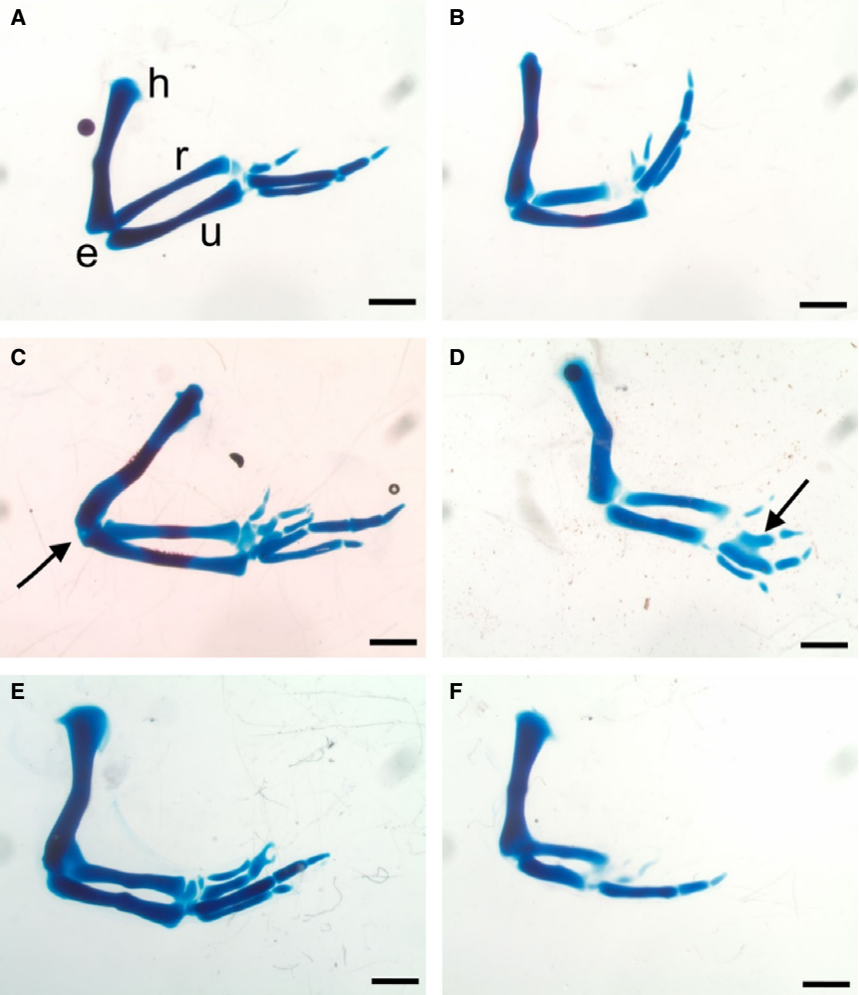
Changes to length : width ratios of humerus, radius and ulna caused by EC23 and ATRA. Whole-mount skeletal preparations of isolated wings are shown from embryos treated with retinoid-soaked beads of 150–300 μm diameter. (A) Normal development of the wing in response to DMSO. (B) Effect of 1 mg mL^−1^ ATRA, producing shortened radius and ulna elements. This also shows a 1123 digit duplication with the two digit 1s fused at the proximal element. (C-F) Effects of 0.01 mg mL^−1^ EC23. (C) Normal length of the humerus, radius and ulna. The arrow indicates a humerus–radius fusion. (D-F) Shortened radius and ulna elements as well as thickened humerus elements. The arrow in (D) indicates a fusion between the 2nd and 3rd digits. (E) One of the wings with a digit duplication designated as ‘other’ with severe thickening of the humerus and shortening of the zeugopod. (F) Severe effects on humerus, radius and ulna lengths. h, humerus; e, elbow; r, radius; u, ulna. Scale bars: 1 mm.

Application of ATRA to the posterior proximal wing can affect zeugopod and autopod elements (Tickle et al. 1985), and element length has been affected in RARα/γ and cyp26 null mice (Lohnes et al. 1994; Yashiro et al. 2004; Dranse et al. 2011). Effects on the proximal wing were also noted with EC23 and ATRA after application of large beads. The frequencies of alterations to more proximal limb elements after application of ATRA and EC23 to anterior limb buds are shown in Tables 5 and 6. Example phenotypes are illustrated in Fig. 7. It can be seen from Table 5 that EC23 and ATRA cause malformation to proximal regions of the wing. The frequency of digit duplication was 65% in EC23-treated wings and 100% in ATRA-treated wings, which was similar to the frequency of effects on zeugopod element size: 69% in EC23-treated wings and 100% in ATRA-treated wings. Both EC23 and ATRA also affected the humerus with a similar frequency. However, in keeping with their differential effects on the digits, EC23 and ATRA showed differential effects on development of the humerus, radius and ulna.

**Table 5:** Effect of EC23 and ATRA on development of humerus, radius and ulna. This shows the frequency of phenotypes and number of examples with a smaller length : width ratio of radius or ulna. Note that embryos exhibiting smaller radius or ulna are both included in the column designated RU change. Some embryos show changes to one or both elements.

Retinoid	Concentration ( mg mL^−1^)	% of embryos (number) surviving after 7 days' treatment with malformations to humerus, radius or ulna cartilages			
		Radius/ulna change	Small radius	Small ulna	Humerus
EC23	0.01	69 (20)	48 (14)	48 (14)	48 (14)
ATRA	1	100 (6)	100 (6)	17 (1)	83 (5)

**Table 6:** Effect of EC23 and ATRA on size of radius, ulna and humerus. Average length: width ratio of the cartilage elements is shown (± S.D.). Significance was tested using an unpaired Student's *t*-test to compare retinoid-treated with DMSO-treated cartilage elements.

Retinoid	Concentration (mg mL^−1^)	Average LW ratio of radius, ulna and humerus 7 days after treatment		
		Radius	Ulna	Humerus
DMSO/untreated	N/A	12.6 (±2.5)	13.2 (±2.2)	11.9 (±1.0)
EC23	0.01	8.6 (±2.5)***	9.2 (±3.1)***	9.4 (±2.3)***
ATRA	1	7.4 (±1.1)***	10.8 (±2.1) *	12.4 (±1.8) NS

* *P* < 0.05,

*** *P* < 0.001.

NS, not significant.

Application of EC23 at HH20 was able to cause a significant decrease in the humerus length : width ratio, which is leftacterised phenotypically by a thickened and shorter humerus. This can be seen by comparison of Fig. 7(A) with Fig. 7(D-F), and Table 6. It can also be seen from Table 5 that ATRA causes alterations to the humerus length : width ratio compared with control embryos at twice the frequency of EC23. However, as shown in Table 6, EC23 causes a statistically significant decrease in humerus length : width ratio compared to controls, whereas ATRA causes a slight increase in this ratio, although this is not statistically significant.

Table 5 shows that 69% of embryos treated with 0.01 mg mL^−1^ EC23 exhibit a change in the length : width ratio of the radius and/or the ulna, whereas the frequency of change to these elements with ATRA is 100%. As can be seen from Fig. 7, ATRA and EC23 can affect the length of both zeugopod elements equally (not shown, Fig. 7E) or one element more than the other (Fig. 7B,D,F). Both retinoids are able to produce a significant reduction in length of both elements, as shown in Table 6. Treatment with 1 mg mL^−1^ ATRA causes a change to the radius (Fig. 7B) or both elements (not shown) in every embryo analysed. There is a notable difference in the frequencies with which EC23 and ATRA affect the different elements. EC23 effects the development of the radius and ulna at the same frequency, whereas ATRA shortened the length of the more anterior radius with a higher frequency than the more posterior ulna (Table 5). In addition, the two retinoids differed in the magnitude of the reductions they produced. ATRA caused a greater shortening of the radius than EC23, whereas EC23 caused a greater shortening of the ulna (Table 6). Effects on the elbow joint were also seen, with fusions between the humerus, radius and ulna, as shown in Fig. 7(C,E).

These data suggest that EC23 and ATRA both affect the entire proximal–distal axis. ATRA has been suggested to affect proximal areas previously (Tickle et al. 1985; Tickle & Crawley, 1988). In the present study this has been shown after application to the anterior wing bud at HH20 rather than the posterior wing bud. There is evidence for a difference in the effects produced by the stable retinoid EC23 compared with ATRA on the zeugopod and stylopod elements. These differences are in the frequency with which changes are seen, the nature and the magnitude of the changes. These proximal effects are consistent with our findings on the induction of the proximal marker *Meis2*, shown in Fig. 5(C).

### Both EC23 and ATRA affect elbow development and cause digit fusions

**Figure 8:**
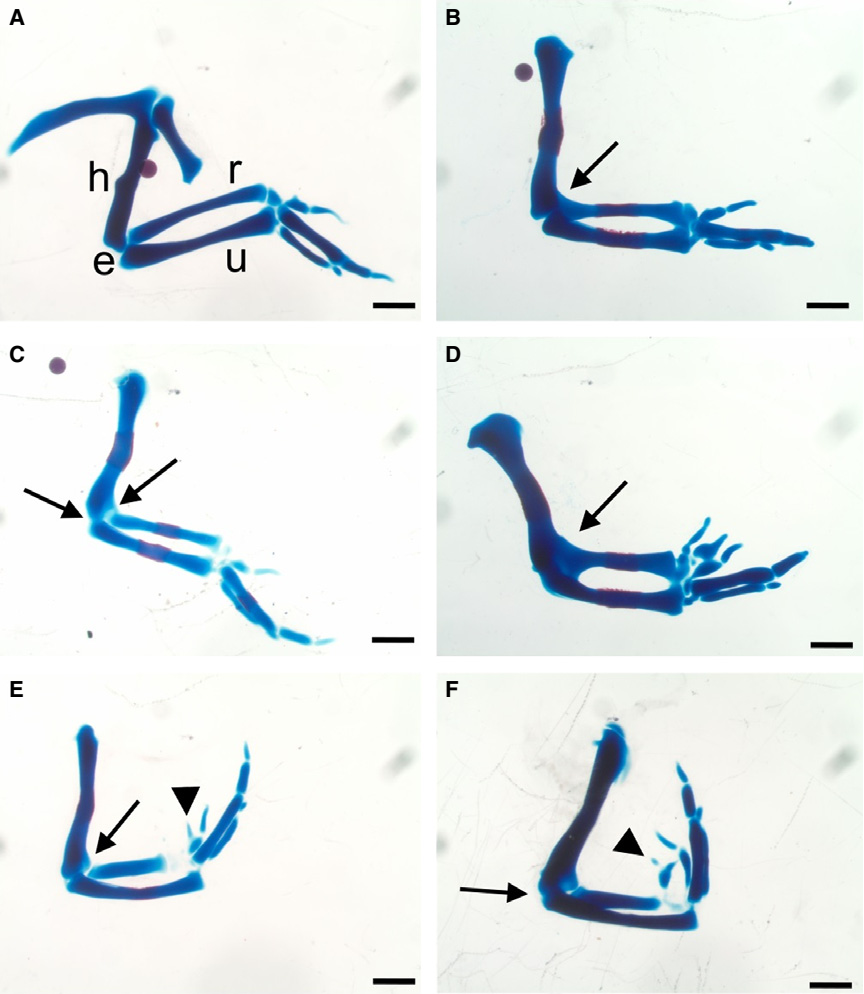
Effect of EC23 and ATRA on elbow development. Whole-mount skeletal preparations of isolated retinoid-treated wings are shown. (A) Untreated wing with normal digit and elbow development. (B-D) Effects of 0.01 mg mL^−1^ EC23 on development of the elbow joint ranging from least to most severe. The arrow in (B) shows a humerus–radius fusion. The arrows in (C) show a mild humerus–radius–ulna fusion. (D) Complete fusion of humerus–radius–ulna (arrow). (E,F) Effect of 1 mg mL^−1^ ATRA on elbow development. (E) Mild humerus–radius–ulna fusion (arrow). F) shows a humerus–ulna fusion (arrow). The arrowheads in (E) and (F) also indicate fusion between the 2nd digits in these duplications. h, humerus; e, elbow; r, radius; u, ulna. Scale bars: 1 mm.

Both EC23 and ATRA can be seen to generate fusions to the elbow joint or between digits during wing development. Table 7 shows the frequencies of elbow phenotypes seen after treatment with EC23 and ATRA. Figure 8 shows representative examples of fusions between different cartilage elements. EC23 has not been observed to cause fusion of the humerus–ulna and ATRA has not been observed to cause fusion of the humerus–radius elements, as shown in Table 7. However, EC23 can fuse humerus–radius, as shown in Fig. 8(B) and ATRA can fuse humerus–ulna, as shown in Fig. 8F. Both EC23 and ATRA can generate fusion of all three elements at varying severity. EC23 0.01 mg mL^−1^ causes fusion of humerus, radius and ulna with varying degrees of severity (*cf*. Fig. 8C,D). As seen from Table 7, EC23 and ATRA cause fusions to developing digits at similar frequencies. Figures 3(D,E), 4(B,E), 7(E) and 8(D) show examples of digit fusions in response to 0.01 mg mL^−1^ EC23, and Fig. 8(E,F) shows fusions after treatment with 1 mg mL^−1^ ATRA. These fusions are largely between cartilage elements of digit 1s, and are similar to radial polydactyly abnormalities described in the clinical literature (Manske & Oberg, 2009).

**Table 7:** Frequency and nature of cartilage element fusion in wing development after treatment with EC23 and ATRA. Note that some examples with digit fusions also exhibited more proximal fusions, and are included in the HR, HU and HRU frequencies.

Retinoid	Concentration (mg mL^−1^)	% of embryos (number) surviving after 7 days treatment with fusions			
		HR	HU	HRU	Digit
EC23	0.01	17 (5)	0	21 (6)	10 (3)
ATRA	1	0	17 (1)	17 (1)	33 (2)

HR, humerus–radius; HU, humerus–ulna; HRU, humerus–radius–ulna.

Because we have observed fusion of elements throughout the proximal–distal axis of the limb we favour this as an explanation of our observations with respect to the digits. An interesting alternative interpretation which we cannot exclude or support without further experiments is that the digit phenotypes listed in the above paragraph arise from bifurcation rather than fusion of elements.

### Synthetic retinoids truncate the limb girdle

Previous research has shown that retinoids can affect the shoulder girdle if applied at HH18-20 in chick embryos and has shown that retinoids caused production of ectopic cartilage or duplication of the coracoid (Oliver et al. 1990). A high concentration of ATRA (10 mg mL^−1^) was required to produce an ectopic cartilaginous spur on the scapula when applied at HH20. We found that naturally occurring and synthetic retinoids were able to affect the scapula when using large beads, but in ways that differed from those which had been described previously (Oliver et al. 1990).

Malformation to the scapula occurs at a similar frequency to digit duplication and alterations of stylopod and zeugopod elements, as shown in Table 8. All embryos treated with 1 mg mL^−1^ ATRA that survived to HH35 exhibited a scapula malformation, whereas 0.01 mg mL^−1^ EC23 caused scapula malformations at a lower frequency. In all cases, scapula phenotypes were seen with digit duplications in response to 1 mg mL^−1^ ATRA. Scapula phenotypes were not always concurrent with digit duplication in response to 0.01 mg mL^−1^ EC23: 24% scapula malformations seen had normal digit development. However, in most cases of EC23 treatment if there was a digit phenotype, there was also a malformation of the scapula: 78% limb phenotypes had a malformed scapula and 67% scapula malformations were seen with digit duplication. In all, 67% scapula phenotypes were seen concomitant with a change in the size of the radius and/or ulna and 52% of scapula malformations were seen with a change in the size of the humerus.

**Table 8:** The effect of EC23 and ATRA on scapula development. The third column shows the frequency of embryos (number) surviving to HH35 with normal scapula on the operated side. The remaining columns show the frequency (number) of embryos with various scapula malformations: reductions, foramen, bending, ectopic cartilage or absent head structure. Note that some examples exhibited more than one of these phenotypes.

Retinoid	Concentration (mg mL^−1^)	% normal at HH35 (number)	% embryos at HH35 (number) with one or more scapula malformations				
		Normal	Reduced	Foramen	Bend	Ectopic cartilage	Absent head
EC23	0.01	24(7)	59 (17)	0	7 (2)	14 (4)	48 (14)
ATRA	1	0	83 (5)	17 (1)	0	17 (1)	67 (4)

**Figure 9:**
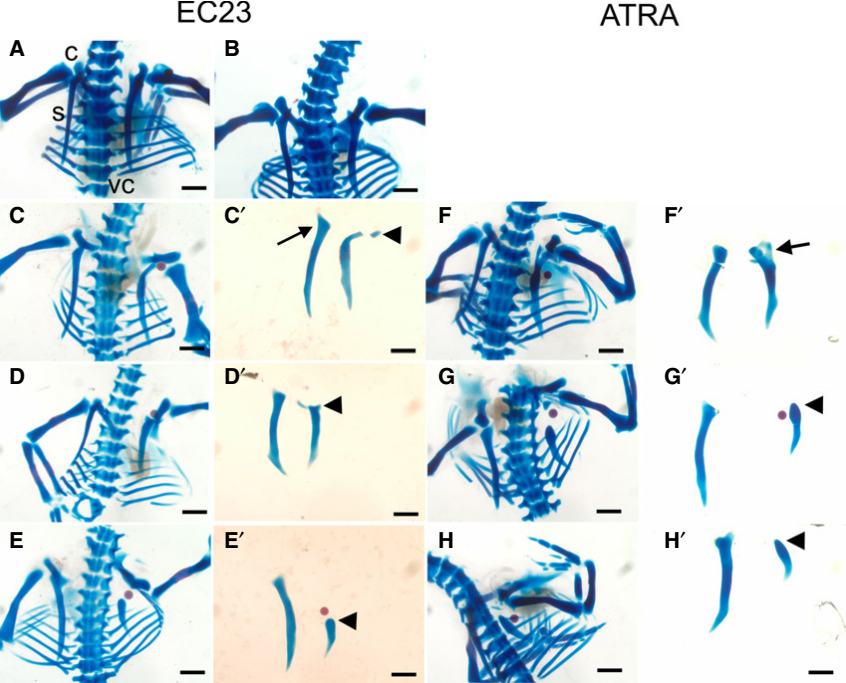
Effect of retinoids on scapula development. Whole-mount skeletal preparations of retinoid-treated torsos and isolated scapulae are shown. (A) DMSO control embryo. (B-E′) Embryos treated with 0.01 mg mL^−1^ EC23 ranging from least to most severely affected. (F-H′) Embryos treated with 1 mg mL^−1^ ATRA, ranging from least to most severe affected. (A-H) Dorsal views of the torso with the operated wing on the right and anterior to the top. (C′-H′) Flat views of both scapulae after dissection: right is from operated side. (C-E) Examples of bending, ectopic cartilage and truncation, respectively. Arrow in (C) indicates the scapular head on the un-operated wing. Arrowheads in (C′-H′) indicate absence of the scapular head on the operated side. Panels F and F′ shows that scapula can develop a foramen (arrow in F′) in the presence of ATRA. (G,G′,H,H′) Reduction to the scapula blade in the presence of ATRA. c, coracoid; s, scapula; vc, vertebral column. Scale bars: 1 mm.

As can be seen from Table 8, the scapula malformations generated ranged in severity. The most frequent scapula malformation seen with either EC23 or ATRA was a shortening of the scapula blade, as shown in Fig. 9. This was often concurrent with absence of the scapula head in EC23 and ATRA-treated embryos, as shown in Fig. 9(C–E′) and Fig. 9(G–H′). There was a range in the severity of this phenotype in both EC23-or ATRA-treated embryos. For example, compare Fig. 9(C′) with Fig. 9(E′) for EC23 and Fig. 9(F′) with Fig. 9(H′) for ATRA.

The least severe phenotypes are those of ectopic cartilage, bending and formation of a foramen, as shown in Figs 9(C,C′ and F,F′). Both EC23 and ATRA can generate ectopic cartilage around the scapula as seen in Fig. 9(C′,G′), consistent with previous literature (Oliver et al. 1990). In contrast, duplication of the coracoid was never seen with EC23 and ATRA in our hands. Only EC23 was seen to have any effect on the coracoid, causing one embryo to develop with a thickened coracoid (not shown). Similarly, significant bending of the scapula blade was only seen in response to treatment with 0.01 mg mL^−1^ EC23, as shown in Fig. 9(C′). The development of a foramen in the scapula was only seen once, in response to 1 mg mL^−1^ ATRA, as shown in Fig. 9(F′). Thus, EC23 and ATRA are shown to affect the limb girdle as well as the entire proximo-distal axis of the wing.

### Both ATRA and EC23 inhibit expression of the scapula precursor marker *Pax1*

The mechanism behind this shortening of the scapula was investigated by analysing the effect of retinoids on *Pax1* expression. *Pax1* has been implicated in scapula development, as the *Pax1* mutant mouse (*undulated*) shows a malformed acromion (head) and spina scapula (Timmons et al. 1994). Its expression pattern has also been documented in mouse and chick development (Timmons et al. 1994; LeClair et al. 1999; Huang et al. 2000). *Pax1* expression in chick has been shown to precede the condensation of scapular cartilage and can be regarded as a marker for scapula development (Huang et al. 2000). Therefore, the mechanism behind the scapula reductions in EC23 and ATRA treated embryos was explored by investigating the effect of these retinoids on *Pax1* expression.

**Figure 10:**
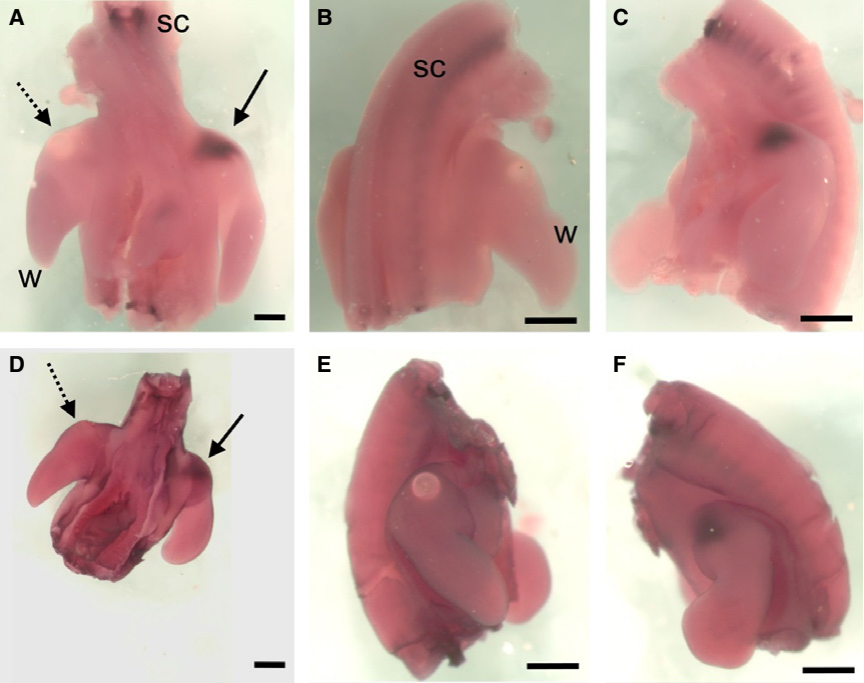
Expression pattern of *Pax1* in response to retinoids. Embryos were treated with retinoid dissolved in DMSO at HH20 and allowed to develop for 48 h. *Pax1* expression was investigated using whole-mount *in situ* hybridisation. (A,D) Ventral views and (B,C,E,F) lateral, dorsal views of chick wings after 48 h of retinoid treatment. (A-C) Embryos treated with 1 mg mL^−1^ ATRA and (D-F) with 0.01 mg mL^−1^ EC23. (B,E) *Pax1* expression is absent in the presence of retinoid. This is also shown by the dashed arrows in (A) and (D). Panels (C) and (F) show normal expression of *Pax1* on the contralateral side of the embryo. This is also shown by the solid arrows in (A) and (D). sc, sclerotome; w, wing. Scale bars: 1 mm.

Figure 10 shows whole-mount *in situ* hybridisation for *Pax1* expression in the wing after 48 h of retinoid treatment with large beads. In untreated wing buds, *Pax1* is seen to be expressed in a domain at the anterio–proximal wing bud (Huang et al. 2000) at HH24–26. Both EC23 and ATRA down-regulate *Pax1* expression in wings treated at HH20 with retinoid-soaked beads. This down-regulation can be complete, as shown in Fig. 10 (*n* = 2/6 ATRA or *n* = 3/5 EC23), or there can be a reduction in *Pax1* levels (*n* = 3/6 ATRA or *n* = 1/5 EC23). These data are consistent with the idea that retinoids cause the scapula phenotypes observed via a down-regulation of *Pax1*.

### Effect of EC23 and ATRA on upper beak outgrowth

**Figure 11:**
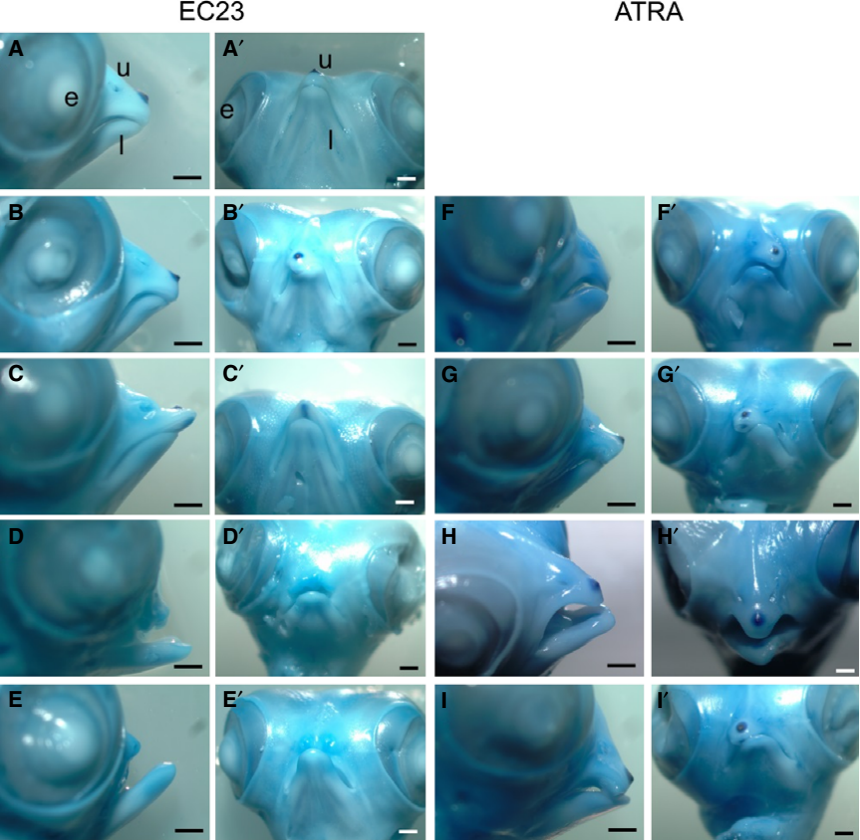
Effects of retinoids on upper beak outgrowth. (A-I) Lateral views of embryos stained with Alcian Blue and (A′-I′) show frontal views of the same embryo. (A,A′) Effect of DMSO on upper beak outgrowth. (B-E′) Phenotypes produced with 0.01 mg mL^−1^ EC23 with increasing severity. (F-I′) Effects of 0.1 mg mL^−1^ EC19 with increasing severity. (B,B′) Asymmetric upper beak outgrowth. (C,C′) Production of an overbite. (D,D′) Reduction in upper beak outgrowth. (E,E′) show complete truncation of the upper beak. (F,F′) Asymmetric upper beak outgrowth with the upper beak skewed to the unoperated side. (G,G′) Asymmetric upper beak outgrowth with the upper beak skewed towards the operated side. (H,H′) Reduction in upper beak outgrowth. (I,I′) Both reduced and asymmetric upper beak outgrowth. e, eye; l, lower beak; u, upper beak. Scale bars: 1 mm.

Retinoids are known to affect the development of the upper beak when applied to the anterior chick wing at HH20 (Tamarin et al. 1984). The whole embryo, including the face, can become exposed to retinoid when it is applied topically to the limb (Thaller & Eichele, 1988). The levels of additional retinoid in the treated limb are found to be 10-fold higher than the rest of the embryo. No overall reduction in embryo size was observed after retinoid treatment, indicating that the facial effects observed reflect a particular sensitivity of these structures to retinoids. The effect of EC23, EC19 and ATRA on upper beak development was compared after application of retinoid to the limb via the large 150–300-μm beads. Table 9 shows the frequencies of facial phenotypes at concentrations chosen for EC23 and ATRA and two concentrations of EC19. EC23 and EC19 generated a range of facial phenotypes (Fig. 11. ATRA generated a mild reduction of upper beak outgrowth in 50% of embryos and the more severe truncated upper beak in 33% of embryos, as previously described (Tamarin et al. 1984).

**Table 9:** The effect of ATRA, EC23 and EC19 on upper beak development in embryos surviving to HH35. Shows the frequency of embryos which survived to HH35 with either normal, asymmetric, reduced, truncated or overbite facial phenotypes. Reduction is leftacterised as decreased upper beak outgrowth whereas truncation is complete absence of the upper beak (see Figure 11 D, D', E, E', H, H'). Numbers are presented as percentages of number surviving to HH35 (number).

Retinoid	Concentration (mg mL^−1^)	Frequency of embryos surviving to HH35 with facial phenotypes% (number)				
		Normal	Assymetric	Reduced	Truncated	Overbite
EC23	0.01	41 (12)	3 (1)	38 (11)	14 (4)	3 (1)
ATRA	1	17 (1)	0	50 (3)	33 (2)	0
EC19	0.01	83 (5)	0	17 (1)	0	0
EC19	0.1	10 (1)	60 (6)	40 (4)	0	0

Exposure to 0.01 mg mL^−1^ EC23 resulted in asymmetric upper beak outgrowth (Fig. 11B,B′) and the production of an overbite (Fig. 11C,C′) at low frequencies. At 0.01 mg mL^−1^, EC23 also caused both reduced and truncated upper beak outgrowth in 38 and 14% of embryos, respectively (Table 9, Fig. 11D,D′,E,E′).

Retinoid EC19 caused mild effects on upper beak outgrowth. At 0.01 mg mL^−1^ EC19, only 17% of embryos exhibited reduced upper beak outgrowth (Table 9 and data not shown). However, at 0.1 mg mL^−1^, EC19 reduced upper beak outgrowth in a similar frequency to EC23 (Fig. 11H,H′, I,I)′. Application of 0.1 mg mL^−1^ EC19 also generated a high frequency (60%) of asymmetric upper beak outgrowth (Fig. 11F,F′,G,G′). The frequency of this phenotype is higher than the frequency of the equivalent seen with 0.01 mg mL^−1^ EC23. It is also notable that although retinoid was always applied to the right wing, the asymmetry can be either towards or away from the source of retinoid (*cf*. Fig. 11F′ with Fig. 11G′). In the most severe phenotype generated by 0.1 mg mL^−1^ EC19, there is both reduced and asymmetric upper beak outgrowth (Fig. 11I,I′). These data indicate that EC23 is more potent than EC19 in its ability to affect upper beak development. EC23 is able to generate more severe phenotypes at a lower concentration than EC19. This is consistent with our findings on limb phenotypes (Fig. 2) and previous reports (Christie et al. 2008).

## Discussion

After applying novel stable synthetic retinoids *in vivo* to chick embryo limb buds, we find that one compound, EC23, has very similar biological effects to *all-trans* retinoic acid. Within the appropriate concentration range, EC23 can be regarded as equivalent to ATRA but with the added benefit of being more stable, both to light and on the basis of its structure, to enzymatic degradation. We found that EC23 is 100–1000-fold more effective than ATRA *in vivo*. Thus, synthetic retinoid EC23 offers the potential to be used as a more consistent experimental tool *in vivo* as well as *in vitro* (Christie et al. 2008). In addition, when higher doses of EC23 are applied, we find that novel phenotypes are produced compared with ATRA; in particular, a novel type of digit duplication was observed. We have also seen effects on the entire proximo–distal axis of the limb with both EC23 and ATRA. These include a novel effect on the pectoral girdle. Differences in outcomes compared with ATRA may be a result of the stability of EC23, or differential receptor activation. A second synthetic retinoid, EC19, differs from EC23 only in the position of the terminal carboxylic acid group. This structure is consistent with the idea that EC19 may be a stable analogue of *13-cis* retinoic acid. We find that EC19 shows a markedly different spectrum of phenotypic effects *in vivo* compared with EC23, as it did *in vitro* (Christie et al. 2008). *13-cis* Retinoic acid has been shown to be present at low concentrations *in vivo*, like *9-cis* retinoic acid, and is increased after the application of excess amounts of ATRA (Horton & Maden, 1995). However *13-cis* retinoic acid has been shown to be less potent than ATRA (Kistler, 1987) and its effects have been attributed to its inter-conversion with ATRA by isomerases (Chen & Juchau, 1998; Ruhl et al. 2001). Thus, EC19 may represent an experimentally useful compound for the study of the effects of *13-cis* retinoic acid in the absence of its conversion to ATRA.

### Potential mechanisms for the differential effects of EC23

Two possibilities may explain the differences in the activities of EC23, ATRA and EC19: differential receptor activation and decreased metabolism *in vivo*. Considering the increased stability and potency of EC23, it is likely that a decrease in its metabolism *in vivo* may explain its alternative action compared with ATRA. The structure of EC23 is similar to that of another synthetic retinoid, TTNPB. TTNPB has been shown to be three orders of magnitude more potent than ATRA (Eichele et al. 1985), which is similar to the potency we find with EC23. TTNPB potency has also been shown to be due to its resistance to metabolism (Pignatello et al. 2002) rather than to changes in binding affinity with retinoid receptors or CRABP (Pignatello et al. 1999). When considering the structure of EC23, it may be predicted that its metabolism by the cyp26 enzymes should be blocked by the replacement of trimethylcyclohexenyl unit of ATRA with a tetramethyl-tetrahydronaphthalene unit, which blocks oxidation at the carbon atoms numbered as 4, 5 and 6 on the structures in Fig. 1 (position 4 in ATRA is the centre which becomes oxidised to give the so-called 4-oxo derivative). There remains the question, however, as to whether the other equivalent positions in EC23 and EC19 could be oxidised, as they are in the proposed oxidative metabolites of ATRA (Topletz et al. 2012), i.e. positions 3, 16 and 18. Assuming that the 4-position is the more readily oxidised in these systems, this would cause ATRA to be inactivated much more efficiently than EC23 by cyp26, as EC23 would be unable to undergo the same oxidation at position 4 due to its geminal-dimethyl substitution at that centre. Also, because EC19 shares a similar structure to EC23, differing only in the position of the terminal carboxylic acid group, it would be just as stable as EC23. Hence, the fact that EC19 shows differential effects compared with both ATRA and EC23 suggests that differential receptor activation is the underlying mechanism for the effects of EC19.

### Retinoids and digit development

ATRA and EC23 produce digit duplications after application to the anterior wing bud at HH20. ATRA has been shown previously to generate duplications (Tickle et al. 1985), as has the synthetic retinoid TTNPB (Eichele et al. 1985), which has some structural similarities to EC23. These early experiments on the limb indicated that retinoids were able to produce changes in the anterior-posterior patterning of the digits. These findings were extended by studies showing that after retinoid treatment there is up-regulation of genes involved in setting up anterio–posterior polarity, for example *hoxb8* after 30 min (Lu et al. 1997; Stratford et al. 1997) and *Hand2* after 20 h (Fernandez-Teran et al. 2000). ATRA application was also shown to up-regulate *shh* after 24 h (Riddle et al. 1993). Shh is known to be the protein secreted by the Zone of Polarising Activity (ZPA) (Riddle et al. 1993) and to be required for patterning of the AP axis of the limb after the stylopod/zeugopod transition (Chiang et al. 2001; Ros et al. 2003). It has, therefore, been proposed that retinoids cause digit duplication through induction of a second ZPA in the anterior mesenchyme. When EC23 is applied via large beads, we have observed the development of multiple digit 1s without duplication of other digits. This phenotype is not observed in response to ATRA (Tickle et al. 1985; Budge, 2010) or TTNPB (Eichele et al. 1985), despite evidence that TTNPB is also resistant to metabolism (Pignatello et al. 2002).

When a fixed quantity of additional retinoid is applied via an ion-exchange bead, the effects of the additional retinoid will eventually be attenuated by metabolism of the added retinoid and growth of the embryo, both of which will reduce retinoid concentrations in tissues. If a retinoid is resistant to metabolism, only the increase in size of the embryo would attenuate retinoid effects. Thus, expression of immediate retinoid targets would be expected to persist for longer in a limb bud treated with a stable retinoid (Pignatello et al. 1999). After applying retinoids via large beads we observed induction of *shh* expression in anterior limb buds after 30 h with ATRA but not EC23. This would mean that *shh* induction occurs later and/or less shh is produced with EC23. Lower levels of shh would then be produced in the anterior limb, compared with ATRA treatment, which would lead to the duplication of only anterior digits (Towers et al. 2008). An example of 11123 pattern digit duplications has been previously described in the literature at low frequency, in an experiment where shh at low concentration was applied to the limb bud via a bead for an extended period (Yang et al. 1997). This would suggest that the phenotype described here with EC23 is in some way shh-dependent and results from a long duration of low level shh activity. In the clinical literature, mutations affecting *shh* regulation have been implicated in radial polydactyly (Lettice et al. 2003; Gurnett et al. 2007). However, the clinical phenotypes involve single duplicated thumbs, not the multiple duplications described here in response to EC23.

In some contexts, retinoids are known to exhibit proliferative effects. Retinoic acid in keratinocytes is known to be able to promote proliferation via an alternative receptor pathway involving PPARβδ and FABP5 (Shaw et al. 2003; Schug et al. 2007). Potentially, this enhanced proliferation could also be occurring in the limb after EC23 treatment. As a result, multiple additional digits are formed, but they all have digit 1 identity associated with low shh levels. In this sense, our anterior digit 1 duplications are occurring under similar conditions to those producing abnormalities in *talpid3* mutants, where shh transduction is blocked but cell proliferation can still occur (Davey et al. 2006; Bangs et al. 2011).

An alternative, non-exclusive possibility relates to the fact that digit 1 is the last to form, as indicated by the initiation of *Sox9* expression in its condensation (Welten et al. 2005). If the presence of EC23 suppresses a number of aspects of limb development, then establishment of digits with identities of 2 and 3 may not be possible in the EC23-affected region of the limb bud, even in the presence of shh. By the time EC23 concentrations fall to levels compatible with digit initiation, it is only possible to form condensations of digit 1 morphology before digits can no longer be initiated at all. In the context of promotional models of digit specification (Towers & Tickle, 2009; Towers et al. 2011), our data would be consistent with there being insufficient time for any promotion of duplicated digits occurring at all after EC23 treatment.

### Retinoids and regulation of cartilage element size

*All-trans* retinoic acid and EC23 have been observed to reduce the length of the humerus, radius and ulna during limb development. They have also been seen to affect these elements differentially: ATRA has less effect on the humerus and ulna than EC23 (Fig. 6, Tables 5 and 6). We have also observed synostosis of different components of the elbow. These findings are consistent with the malformations described in mice, zebrafish and man in situations in which cyp26b1 function is impaired (Yashiro et al. 2004; Laue et al. 2008, 2011; Spoorendonk et al. 2008). *Cyp26b1* inactivation would be predicted to give rise to increases in endogenous retinoid levels.

Previously, retinoids have been shown to down-regulate fgfs and distally expressed genes in limb development, causing cell identity to become proximalised (Mercader et al. 2000). The effect of both EC23 and ATRA on cartilage element length may be due to transient down-regulation of the AER and decreased proliferation of progenitors. The effects on the ulna are consistent with a deficiency of *shh* during its development (Chiang et al. 2001; Ros et al. 2003), which would be consistent with our observations of a delay in *shh* induction.

The shortened and thickened zeugopod elements we have seen are similar to those produced by over-expression of *Meis1* (Mercader et al. 1999, 2009). This would be consistent with up-regulation of the proximal marker, *Meis2*, seen in treated limb buds, suggesting that retinoids are proximalising limbs via induction of *Meis1* and *Meis2*. We have unpublished data from a microarray analysis (data not shown) of retinoid-treated limbs indicating that, in addition to *Meis2*, there is also up-regulation of *Meis1* in ATRA-and EC23-treated limbs compared with DMSO. *Meis1* up-regulation is consistent across all retinoid-treated samples, although the level of variation between replicate samples means that the observation does not pass filtering criteria for significantly altered target genes. The enhanced ability of EC23 to induce proximal markers compared with ATRA may mean that it represents a useful tool for exploring the role of retinoids in the establishment of proximal identity in limb buds.

### Effect of retinoids on scapula development

EC23 and ATRA cause shortening to the scapula blade, and absence of the scapula head is seen at high frequency. Both ATRA and EC23 down-regulated *Pax1* expression. *Pax1* has been proposed to be a marker of scapula-forming cells and is expressed during the extension of the scapula blade (Huang et al. 2000). *Pax1* null mice (*undulated*) have been shown to exhibit malformations in the shoulder girdle (Timmons et al. 1994). Increased BMP signalling has been implicated in scapula malformation and inhibition of *Pax1* expression between HH19 and 25 (Hofmann et al. 1998). Injection of Noggin-expressing cells at varying somite levels between HH20 and 22 caused lack of scapula blade development in the corresponding somite level (Wang et al. 2005). ATRA and EC23 may truncate scapula development via perturbation of BMP signalling. *Pbx1* has also been implicated in the control of scapula development, given its expression pattern and knockout mice exhibiting reduction of the scapula blade (Selleri et al. 2001; Capellini et al. 2010). Interestingly, *Pbx1* has also been documented as a retinoid target during limb development (Qin et al. 2002). Alteration in the expression of these two transcription factors may be a mechanism by which ATRA and EC23 affect scapula development.

### Retinoids and upper beak outgrowth

*All-trans* retinoic acid and EC23 can be seen to reduce or truncate upper beak outgrowth. These results are consistent with those reported for ATRA (Tamarin et al. 1984). Upper beak outgrowth and dorso–ventral polarity are controlled by a region called the frontonasal ectodermal zone (FEZ), which develops at HH20 in chick embryos. *Shh* and *fgf8* are essential for outgrowth and morphogenesis of the upper beak, as removal of the FEZ or inhibition of *fgf8* at HH17 causes truncation of the upper beak (Hu & Marcucio, 2009) and excess shh causes duplications or widening of the upper beak (Hu & Helms, 1999). Inhibition of shh signalling has been documented to cause truncation, but it has been shown that the epithelium can re-specify itself after FEZ excision at HH20 to produce normal upper beak development (Hu & Helms, 1999). Retinoids may cause decreased upper beak outgrowth by interfering with the FEZ. Consistent with this, s*hh* expression is absent from 30 h after ATRA treatment in the developing frontonasal mass (FNM), whereas *fgf8* is unaltered (Helms et al. 1997). ATRA is known to have an antagonistic relationship with *fgf8* in the limb bud and other areas (Mercader et al. 2000). Excess retinoid may cause incorrect levels of *fgf8*, which has been shown to result in decreased proliferation and outgrowth of the FNM (Song et al. 2004). To support this, it has been shown that after excess shh is applied to the FNM, there is an increase in *Raldh2* expression and a concurrent decrease in *fgf8* expression which also leads to truncation of the upper beak (Hu & Marcucio, 2009). ATRA has been shown to decrease *Msx* expression in the developing facial processes, which can also lead to decreased outgrowth of the FNM (Brown et al. 1997; Song et al. 2004).

EC19 can also produce facial phenotypes (Fig. 10, Table 9); however, those observed appear to be distinct from those produced by ATRA or EC23 in that the outgrowth of the FNM is reduced or asymmetric. This may be a consequence of its potential activity as an equivalent of *13*-*cis* retinoic acid that cannot be converted to ATRA.

## Conclusions

We have observed that EC23 and EC19 exhibit differential effects on toxicity, limb and craniofacial development *in vivo*. EC23 mimics ATRA to an extent in the production of digit duplications, shortening of the cartilage elements and malformation of the scapula but also generates a novel digit duplication in the production of multiple digit 1s. EC19, however, is never seen to generate digit duplications at the higher concentrations tested herein. EC23 mimics the effects of ATRA on upper beak outgrowth, whereas EC19 causes far milder effects: reductions and asymmetrical outgrowth. We have demonstrated that novel stable retinoids are biologically active and can be used to uncover new aspects of retinoid function *in vivo*. As a result, EC23 and EC19 may represent useful new tools to investigate the mechanisms underlying congenital abnormalities in mammals, including man.
